# On Large Doses of Sub-Nitrate of Bismuth in Gastro-Intestinal Affections

**Published:** 1849-10

**Authors:** 


*On Large Doses of Sub-Nitrate of Bismuth in Gastro-Intestinal Affections.* By M. Monneret.

M. Monneret states that he has been for some years engaged in experimenting upon the medicinal qualities of this substance, and that he finds that, given in far larger doses than are supposed to be permissible, it is of the greatest value in gastro-intestinal affections, especially those attended with fluxes.

*Simple Diarrhœa.* It is especially in the diarrhœa of infants, resulting from imperfect lactation, that he has found it so very useful, large doses curing such in a few days. So, too, after weaning, and the injudicious diet so often adopted, the bismuth, in doses of three teaspoonfuls a day, soon removes all symptoms of disordered digestion, especially the serous diarrhœa so often produced in these cases. Simple diarrhœa is of much rarer occurrence in the adult; still, examples are met with in which we cannot attribute it to either phlegmasia or ulceration of the intestines, as in the granular disease of the kidneys, in chlorotic women, in women become anæmic from cancerous degeneration of the uterus, and in some cases of chronic disease of the heart, or in commencing phthisis. Those cases in which the diarrhœa is serous, and results from an atonic state of the gastro-intestinal membranes, best yield to this medicine. It should be given in gradually increasing quantities from two drachms to twelve or more per diem.

*Diarrhœa symptomatic of Intestinal Lesion.* In spite of the presence of symptoms indicative of inflammatory action, the author has frequently administered the bismuth to children with great success. So, too, he has found it very useful in some subjects of phthisis suffering from obstinate colliquative diarrhœa, and even in the diarrhœa of typhoid fever, when the intestinal canal was certainly the seat of very numerous ulcerations. In this last case its utility is not very permanent; but it has the advantage of inducing the tolerance of articles of diet or medicine, which without it would be rejected.

*Cholerine.* The frequency with which gastro-intestinal disturbance precedes true cholera is well known, and the durability of its suppression is evident; and to this end large doses of bismuth succeed better than any other means. It is most suitable in the cases in which with the diarrhœa there are nausea, vomiting, gastralgia, colicky pains, borborygmi, and anorexia. The cases are innumerable, in which the author has been enabled rapidly to relieve these symptoms without any aid from opium, by giving eight or ten drachms per diem. The only inconvenience resulting, is a certain amount of constipation.

*Gastralgia.* Various observers have already admitted the great utility of this substance, both in idiopathic gastralgia, and in the forms dependent upon hysteria, hypochondriasis, chlorosis, &c., &c.; but it has frequently failed in the hands of others, by being given in doses of two or three scruples per diem, in place of twelve or fifteen drachms; and it is taken with such ease, that the patients voluntarily continue its employment after the pain has ceased, until the gastro-intestinal functions are thoroughly re-established. The idiopathic forms of gastralgia, so often met with in sedentary or literary persons, and in those suffering from depressing emotions, or from abuse of alcohol or coffee, are those which are most successfully combatted. It has often, too, enabled the stomach to bear those tonic aliments, whose slow and painful digestion, accompanied by cephalalgia, drowsiness, and pain in the back and limbs, is so often observed in chloro-anæmic patients. This medicine only produces a temporary relief in gastralgia, dependent upon organic disease of the stomach, except when this viscus secretes a large quantity of acid fluid.

*Vomiting.* Vomiting may depend upon a simple gastric neurosis, and then the bismuth can be very usefully employed. It is also useful in the vomiting of pregnancy, and that accompanying dysmenorrhœa; but its efficacy is less certain than in affections of the gastro-intestinal tube, and is never so great as when diarrhœa, colic, and flatus are present. From whatever cause pain manifests itself during digestion, we may relieve it by mixing the sub-nitrate freely with the articles of food.

*Administration and action.* It should be given in powder, and best so with the first spoonful of broth or gruel. It excites no disgust, especially if placed between two bits of bread soaked in broth. Children take it readily with milk or ptisans. The author has never given less than from two to three drachms per diem, nor more than twenty, and he has never observed the slightest inconvenience from these large doses; and it is his custom to give it to the children in his hospital by spoonfuls or tablespoonfuls, without observing more exactitude, so innocuous is it. So imperfectly is this fact known, that the chemist hesitates in preparing his prescriptions in which these large doses are ordered. The author cannot conceive why this substance was ever set down as an irritant poison, as he has never found the slightest irritation from the largest doses given to either the healthy or the sick; and post-mortem examination proves that, beyond patches of black discoloration, it produces no effect on the mucous membrane, the consistence of this remaining quite normal. The action of the substance on the canal seems to be quite negative, that is to say, the anormal symptoms for which it was prescribed quickly disappear. Great attention has failed also to detect any marked effect upon any other part of the system. Perhaps the urine is somewhat increased, and the pulse becomes slower, but this is probably due to the relief of the gastro-intestinal affection. The action of bismuth is then purely a *local* one; and this local action, so far from being an irritant one, as so commonly stated, diminishes the activity of the phenomena of which the mucous membrane is the seat. It is not to be supposed, however, that this inert substance can act actively and directly upon the canal, as a soluble body after absorption does do; and it is probable that it acts merely as an inert body, just as charcoal might do, mechanically protecting the over-excited secretory organs and papillæ of the nerves from the too immediate conflict of the fluids, and especially of aliments. By its mechanical apposition, it limits the amount of exhalation. The allegation of its anti-spasmodic properties has solely risen from observing the diminution of the symptoms, without considering whether this might not arise negatively from the preservation of the gastro-intestinal surface from the causes of irritation. In this way we might call darkness an anti-spasmodic, because it allows the repose of the retina, and the subdual of the irritation which gives rise to photophobia.—*Gazette Medicale*, Nos. 15, 16.

Of the utility of bismuth in smaller doses in gastric affections, we have almost daily proof; but the doses we are in the habit of employing are so very much smaller than those recommended by M. Monneret, that their beneficial action can scarcely be explained upon the mechanical theory he advocates, which is, however, admirably suited to the explanation of the *modus operandi* of the very large doses he employs. As somewhat confirmatory of his view, we may mention that M. Belloc, in a recent communication to the Academie, makes mention of the very great benefit derivable from the employment of *charcoal* prepared from poplar shoots, in gastro-intestinal affections. It rapidly subdues pain, facilitates digestion, excites the appetite, and enables medicines that the alimentary canal would not otherwise tolerate to be easily borne.—See *Rev. Med.-Chir.*, t. v., pp. 75-86.

